# Dr. Benjamin Wilson

**Published:** 1911-11

**Authors:** 


Dr. Benjamin Wilson, P. & S., 1863, surgeon of U. S. Volunteers during Civil War, died at Rochester, August 13, aged 71.

